# Immuno-pathological studies on broiler chicken experimentally infected with *Escherichia coli* and supplemented with neem (*Azadirachta indica*) leaf extract

**DOI:** 10.14202/vetworld.2016.735-741

**Published:** 2016-07-16

**Authors:** Vikash Sharma, K. K. Jakhar, Swati Dahiya

**Affiliations:** 1 Department of Veterinary Pathology, Lala Lajpat Rai University of Veterinary & Animal Sciences, Hisar, Haryana, India; 2 Department of Veterinary Microbiology, Lala Lajpat Rai University of Veterinary & Animal Sciences, Hisar, Haryana, India

**Keywords:** broiler chicken, *Escherichia coli*, immune response, neem leaf extract

## Abstract

**Aim:**

The present study was conducted to evaluate the effects of neem leaf extract (NLE) supplementation on immunological response and pathology of different lymphoid organs in experimentally *Escherichia coli* challenged broiler chickens.

**Materials and Methods:**

For this study, we procured 192-day-old broiler chicks from local hatchery and divided them into Groups A and Group B containing 96 birds each on the first day. Chicks of Group A were supplemented with 10% NLE in water, whereas chicks of Group B were not supplemented with NLE throughout the experiment. At 7^th^ day of age, chicks of Group A were divided into A1 and A2 and Group B into B1 and B2 with 54 and 42 chicks, respectively, and chicks of Groups A1 and B1 were injected with *E. coli* O78 at 10^7^ colony-forming units/0.5 ml intraperitoneally. Six chicks from each group were sacrificed at 0, 2, 4, 7, 14, 21, and 28 days post infection; blood was collected and thorough post-mortem examination was conducted. Tissue pieces of spleen and bursa of Fabricius were collected in 10% buffered formalin for histopathological examination. Serum was separated for immunological studies.

**Result:**

*E. coli* specific antibody titer was significantly higher in Group A1 in comparison to Group B1. Delayed-type hypersensitivity response against 2,4 dinirochlorobenzene (DNCB) antigen was significantly higher in Group A1 as compared to Group B1. Pathological studies revealed that *E. coli* infection caused depletion of lymphocytes in bursa of Fabricius and spleen. Severity of lesions in Group A1 was significantly lower in comparison to Group B1.

**Conclusion:**

10% NLE supplementation enhanced the humoral as well as cellular immune responses attributed to its immunomodulatory property in experimentally *E. coli* infected broiler chicken.

## Introduction

Avian colibacillosis is one of the important bacterial diseases of poultry caused by *Escherichia coli*, producing considerable morbidity and mortality and associated with heavy economic losses to the poultry industry and is an association with various disease conditions as a primary pathogen or secondary pathogen [1]. Although *E. coli* is a commensal organism of the intestinal tract of poultry but under certain adverse conditions such as poor ventilation, overcrowding, and immunosuppression, it turns pathogenic [2]. Frequent association of *E. coli* with various immunosuppressive diseases like Gumboro disease and in young birds, in which immune system is not fully developed has been found. The O78:K80, O1:K1, and O2:K1 are most commonly found serotypes of *E. coli* in domestic poultry associated with colibacillosis. These *E. coli* strains are usually resistant to chloramphenicol, cefradine, tetracyclines [3,4], β-lactam antibiotics, sulfonamides [5,6], and aminoglycosides [4,6].

Neem tree is a rapidly growing evergreen tree and has medicinal as well as nutritive value for poultry. Chemicals such as azadiractin, nimbin, nimbindin, and quercetin are found in different parts of neem [7-9] having antioxidant, antifungal, antimicrobial, antihelminth, insecticidal, antiprotozoal, and spermicidal properties [10,11]. In addition, neem also has role in improving the immune system of the body. Increase in antibodies against infectious bursal disease and Newcastle disease viruses had been reported by incorporation of neem in poultry feeds [12]. It has been reported that neem leaves addition in feed of broiler chicken has potentiating effects on production of antibody against Newcastle disease and infectious bursal disease viruses [13]. Neem extracts have been shown to possess antibacterial, antifungal, potent antiviral, and anticancerous properties [14-17]. An animal with a good immune system is considered generally able to overcome many pathogenic infections to a larger extent. The need for enhanced immunity is very relevant in any livestock and poultry industry.

Therefore, the present study was conducted to observe the effect of neem leaf extract (NLE) supplementation on the immune response of broiler chickens experimentally infected with *E. coli* infection.

## Materials and Methods

### Ethical approval

We conducted the experiment after approval from the Institutional Animal Ethics Committee.

### Experimental design

For this study, we procured 192-day-old broiler chicks from a local hatchery and divided them into Group A and Group B containing 96 birds each on the first day. Chicks of Group A were supplemented with 10% NLE in water, whereas chicks of Group B were not supplemented with NLE throughout the experiment. At 7^th^ day of age, chicks of Group A were divided into A1 and A2 and Group B into B1 and B2 with 54 and 42 chicks, respectively, and chicks of Groups A1 and B1 were injected with *E. coli* O78 at 10^7^ colony-forming units (CFUs)/0.5 ml intraperitoneally. Blood was collected from six chicks of each group at 0, 2, 4, 7, 14, 21, and 28 days post infection for immunological studies. After collection of blood, the birds from each group were sacrificed at the above-mentioned time intervals and thorough post-mortem examination was conducted. Tissue pieces of different lymphoid organs showing lesions were collected for histopathological examination in 10% buffered formalin. The serum samples from the infected groups were analyzed for antibody titer against *E. coli* infection using indirect enzyme-linked immunosorbent assay (ELISA) [18].

### Preparation of NLE

Neem leaves collected from the campus of CCS Haryana Agricultural University, and the leaves were dried in the shade. The dried leaves were then powdered, and 100 g of neem leaves powder was boiled in 1 L of water for 15 min. The extract obtained after straining it, and the volume was adjusted to 1 L by adding drinking water [19].

### Preparation of E. coli inoculums

O78 serotype of *E. coli* isolated from natural cases was inoculated into brain heart infusion broth (BHIB) and incubated at 37°C for 24 h. Viable count of *E. coli* organism per ml of BHIB was determined by surface spread method [20]. Serial 10-fold dilutions of the above culture were prepared in sterile phosphate buffer saline, and 0.1 ml of each dilution was pipetted onto three MacConkey’s Lactose Agar (MLA) plates. The inoculum on the plates was spread, and these plates were incubated at 37°C for 24 h. The average count of three plates of particular dilution having colonies in the range of 30-300 was calculated. This bacterial count for particular dilution was made in 0.1 ml, the inoculum used for each dilution. Then, the viable count per ml was determined which was considered as CFUs of the *E. coli*. The infective dose at 10^7^ CFU of *E. coli*/0.5 ml was prepared for the experiment as *E. coli* inoculums [21].

### Preparation of E. coli antigen

Stock culture of *E. coli* O78 was grown on BHIB for 24 h at 37°C. The growth so obtained was harvested in sterile normal saline solution (NSS) by centrifugation at 2000× *g* for 30 min. The culture was checked for purity on MLA. The pellet was resuspended and washed three times in sterile NSS at 2000×*g* for 30 min. The cell suspension was sonicated for 3 cycles of 1 min duration and at 1 min interval on ice in a labsonic 1510 sonifier. The sonicated bacterial cell suspension was termed as *E. coli* O78 sonicated antigen that was used for coating the ELISA plates. It was stored in aliquots at −20°C until use.

### Delayed-type hypersensitivity (DTH) response

DTH response was observed by DTH skin test using the method of Tiwary and Goel [22]. Di nitro chloro benzene (DNCB) was used as an eliciting antigen. At the age of 22 days, 0.25 ml of 1% DNCB which was prepared in a vehicle consisting of acetone and olive oil (4:1) mixture was applied on a relatively featherless elliptical area on left side of the abdomen of the birds in each group. Before applying the DNCB solution, the area was first cleaned with ethyl alcohol. The right side of the abdomen was left as control where same amount (0.25 ml) of only the vehicle (acetone and olive oil) was applied. On day 13^th^ post sensitization, 0.25 ml of 0.1% DNCB was applied as eliciting antigen on the left side of the abdomen of each sensitized bird. For control purpose, 0.25 ml of the mixture of acetone and olive oil was painted on the right side. Vernier calipers were used for measuring the skin thickness at 0 and 24 h post challenge of the eliciting antigen. Baseline skin thickness of the same site before the challenge was deducted from the thickness measured at 24 h post challenges of the eliciting antigen to obtain the increment in mean skin thickness.

### Gross pathology

Thorough post-mortem examination of the chicks sacrificed or died naturally during the experiment was conducted to observe gross lesions in lymphoid organs if any.

### Histopathology

Paraffin embedding technique was used for processing the formalin fixed tissues. The tissues were properly trimmed, washed in running tap water, dehydrated in graded ethyl alcohol, cleared in cedar wood oil, and embedded in paraffin wax (melting point 60-62°C). Sections of 4-5 µ thickness were cut using semiautomatic microtome and stained with hematoxylin and eosin. To study the cellular reaction of DTH, a portion of the skin was collected in 10% buffered formalin and processed by hematoxylin and eosin staining method [23].

### Lesion score

The specific gross lesion score (GLS) and histopathological lesion score (HLS) in different experimental groups were calculated for different lymphoid organs/tissues at scale of 0-4 as detailed below:

- 0=No lesion
- 1=Mild lesions
- 2=Moderate lesions
- 3=Moderately severe lesions
- 4=Severe lesions

Percent mean gross and histopathological lesions were calculated as per method described by Witter [24] with slight modifications with the following formula.


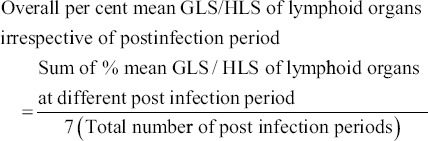



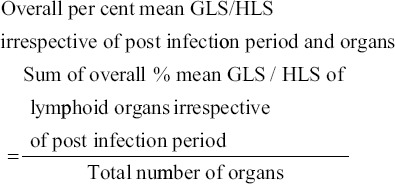



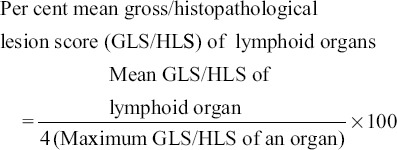


#### Protective effect

Percent protective effect due to NLE supplementation in *E. coli* infected chickens was calculated on the basis of GLS and HLS as per method of Witter [24] using following formula:


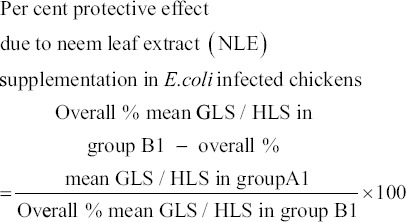


### Statistical analysis

Statistical analysis was performed using analysis of variance technique through *post-hoc* - Duncan LSD Alpha (0.05) using SPSS 16.0 version software, and standard errors of means were used to interpret the results.

## Results

### Antibody titer

**Figure-1:**
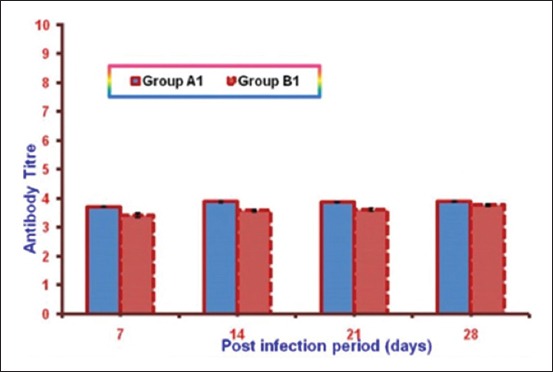
Mean reciprocal log_10_ antibody titer against *Escherichia coli* infection in both the infected groups at different intervals.

Mean reciprocal log_10_ antibody titer against *E. coli* infection in both the infected groups is illustrated in Figure-1. It was observed that mean antibody titer in Group A1 was significantly higher as compared to Group B1 throughout the experiment.

### DTH response

**Figure-2:**
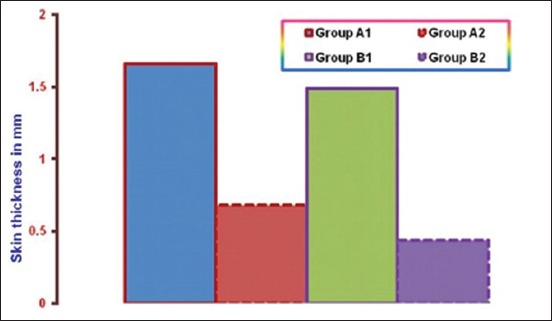
Mean increment in skin thickness due to delayed-type hypersensitivity response in different experimental groups.

**Figure-3:**
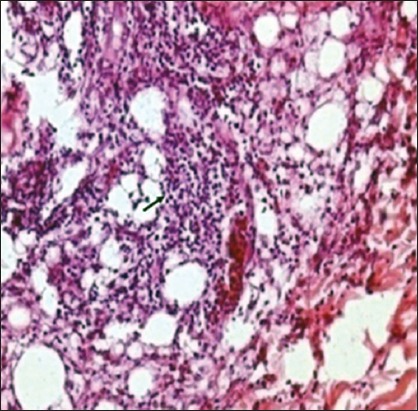
Skin (Group A1): Delayed-type hypersensitivity reaction characterized by large number of mononuclear cells (arrow) and heterophils in dermis (H and E, ×400).

**Figure-4:**
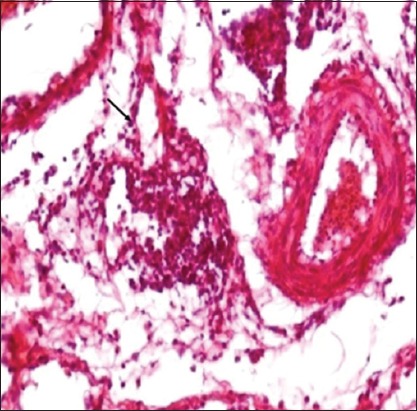
Skin (Group B1): Delayed-type hypersensitivity reaction characterized by fewer mononuclear cells (arrow) and heterophils in dermis (H and E, ×400).

The mean increment in skin thickness of birds due to DTH response in different experimental groups is illustrated in Figure-2. Gross examination of skin showed varying degree of edema, swelling and induration at the site of DNCB application, but no such change was noticed at the site where only olive oil was applied. Measurement of the swelling revealed that mean increase in skin thickness due to DTH response significantly increases in both the infected groups as compared to non-infected ones and was significantly higher in NLE supplemented groups as compared to non-supplemented groups. The increase in skin thickness in the pure infected group was less as compared to NLE supplemented infected group. Histopathological studies of the skin sections from the test site in different experimental groups revealed infiltration of heterophils and mononuclear cells in the dermis (Figures-3 and 4). The cellular infiltration was intense in NLE supplemented infected group, moderate in infected group, less intense in NLE supplemented group, whereas mild in the control group. No cellular reaction was noticed from the skin that was applied with only olive oil. DTH response against DNCB antigen was considerably higher in NLE supplemented groups in compared to non-supplemented groups in the present study, indicating enhanced cellular immune response due to NLE supplementation.

### Gross lesions in lymphoid organs

**Figure-5:**
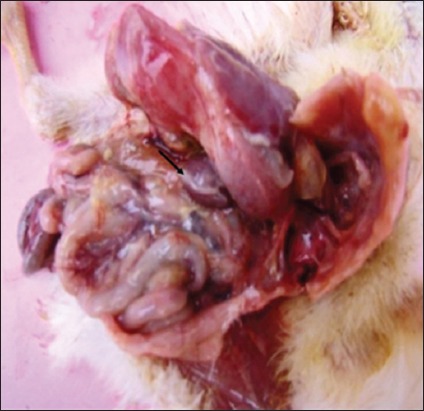
Bird from Group B1 at 7-day post infection showing layer of fibrin (arrow) on spleen.

Gross lesions in the non-supplemented infected group were noticed from 2-day post infection (DPI). On 2 DPI, there was congestion in spleen, whereas on 4 DPI, there was congestion as well as enlargement of spleen. On 7 DPI, there was congestion, enlargement of spleen along with deposition of fibrin on the surface (Figure-5) and bursa of Fabricius was atrophied. On 14 DPI, bursa of Fabricius was atrophied and spleen was congested, and on 21 DPI, bursa showed only mild congestion. In NLE supplemented infected group, similar changes were observed but were of mild nature.

### Histopathological lesions in lymphoid organs

**Figure-6:**
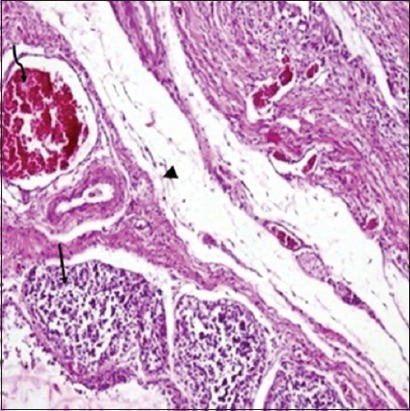
Bursa of Fabricius (Group B1: 2-day post infection): Congestion (curved arrow), sero-fibrinous exudation (arrowhead) in capsule and mild depletion of lymphocytes (arrow) in bursal follicles (H and E, ×200).

**Figure-7:**
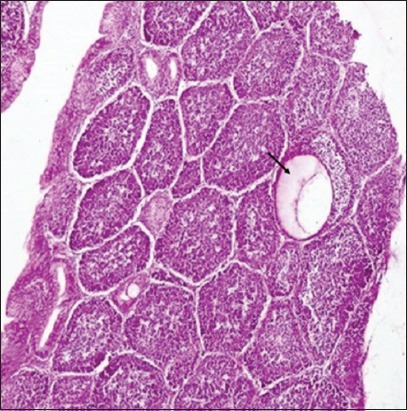
Bursa of Fabricius (Group B1: 4-day post infection): Cystic structure (arrow) formation in bursal follicle (H and E, ×100).

**Figure-8:**
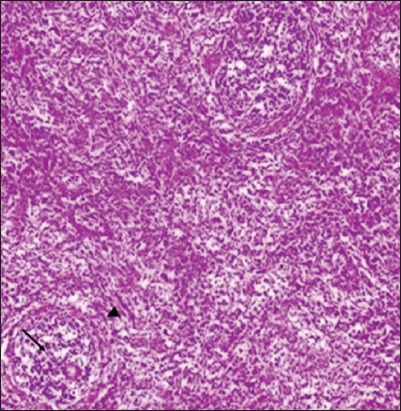
Spleen (Group B1: 7-day post infection): Depletion of lymphocytes in white pulp (arrow), reticuloendothelial cell hyperplasia (arrow head) (H and E, ×200).

Histopathological studies revealed that in non-supplemented infected group on 2 DPI, mild depletion of lymphocyte in white pulp was observed in spleen and bursa of Fabricius revealed marked depletion of lymphocytes, congestion, and sero-fibrinous exudation in capsule (Figure-6). On 4 DPI, spleen showed sero-fibrinous exudation along with leukocytic cells infiltration in capsule as well as depletion of lymphocytes and coagulative necrosis along with reticulo endothelial cell hyperplasia. Bursa of Fabricius exhibited severe lymphocytic depletion leading to formation of cystic structure in bursal follicles (Figure-7). On 7 DPI, depletion of lymphocytes in white pulp along with proliferation of reticuloendothelial cells was observed in spleen (Figure-8). In bursa of Fabricius, there was severe depletion of lymphocytes and small vesicle formation in some bursal follicles. On 14 DPI, spleen revealed severe congestion, hemorrhages, depletion of lymphocytes, and necrosis in white pulp along with reticuloendothelial cells proliferation. Bursa of Fabricius revealed severe atrophy of bursal follicles along with depletion of lymphocytes. On 21 DPI, both bursa of Fabricius and spleen revealed depletion of lymphocytes. In NLE supplemented infected group on 2 DPI, there was depletion in the white pulp in spleen. Bursa of Fabricius revealed mild depletion of lymphocytes in the medullary area of the follicles. On 4 DPI, spleen revealed small necrotic areas along with depletion of lymphocytes in the white pulp. In bursa of Fabricius, depletion of lymphocytes was evident along with mild fibrinous exudate in interfollicular tissue. On 7 DPI, spleen showed depletion of lymphocytes and reticuloendothelial cells proliferation, whereas bursa of Fabricius revealed depletion of lymphocytes in number of follicles. On 14 DPI, there was depletion of some lymphocytes in the white pulp in spleen and in the follicles of bursa of Fabricius. On 21 DPI, secondary follicles were observed along with reticuloendothelial cells hyperplasia in spleen indicating recovery. Colibacillosis in the present study caused severe depletion of lymphocytes in spleen and bursa of Fabricius. In bursa of Fabricius congestion and sero-fibrinous exudation in capsule was also observed in some cases. These lesions in NLE supplemented infected group were of lesser intensity at different intervals as compared to those observed in the non-supplemented infected group.

### Lesion scores and percent protective effect due to NLE supplementation

Colibacillosis specific mean GLS and HLS of spleen and bursa of Fabricius were considerably lower in NLE supplemented infected group (A1) as compared to non-supplemented infected Group B1. Similarly, overall % mean lesion scores irrespective of post-infection period and organs in different experimental groups were also lower in supplemented infected group as compared to the non-supplemented infected group. % protective effect on gross and histopathological lesions of *E. coli* infection due to supplementation of NLE was 42.55% and 42.78%, respectively (Table-1).

**Table-1:** Overall percent mean GLS and HLS in *E. coli* infected groups and percent protective effect due to NLE supplementation in chickens.

Lesion score	Groups	Overall percent mean scores in different organs irrespective of post-infection period		Overall percent mean lesion scores irrespective of post-infection period and organs	Percent protective effect due to 10% NLE supplementation

		Spleen	Bursa of Fabricius
GLS	Group A1	22.6	16	19.3	42.55
	Group B1	38.07	29.14	33.6	
HLS	Group A1	26.78	20.82	23.8	42.78
	Group B1	45.21	38	41.6	

GLS=Gross lesion scores, HLS=Histopathological lesion scores, NLE=Neem leaf extract, *E. coli=Escherichia coli*

## Discussion

Mean antibody titer in NLE supplemented infected group (A1) was significantly higher as compared to non-supplemented infected group (B1) throughout the experiment. These results suggest enhancement of humoral immune response due to NLE supplementation against *E. coli* infection. These findings of the present study are in accordance with Kwawukume *et al*. [25] and Zahid *et al*. [13]. DTH response against DNCB antigen was considerably higher in NLE supplemented groups as compared to control group in the present study, indicating enhanced cellular immune response due to NLE supplementation. More or less similar results have been documented by the other workers in chickens [19,25] as they reported enhanced cell mediated immune response in NLE supplementation. Other workers have also reported similar gross lesions [4,21,26,27] and histopathological lesions [21,27,28] in spleen and bursa of Fabricius in colibacillosis. These lesions in Group A1 were of lesser intensity at different intervals as compared to those observed in non-supplemented infected group.

## Conclusion

Supplementation of 10% NLE in water in experimentally *E. coli* infected broiler chickens reduced the severity of the lesions in spleen and bursa suggesting the protective role of NLE in limiting the depletion of lymphocytes, and second, enhanced the cell-mediated as well as humoral immune response suggesting its immunomodulatory effect.

## Authors’ Contributions

VS and KKJ designed and planned the research experiment. VS performed the research experiment. SD helped in conducting experiment. All authors read and approved the final manuscript.
